# Long-Term Effects of COVID-19 on Women’s Reproductive Health and Its Association with Autoimmune Diseases, Including Multiple Sclerosis

**DOI:** 10.3390/jcm14093057

**Published:** 2025-04-29

**Authors:** Efthalia Moustakli, Sofoklis Stavros, Theologos M. Michaelidis, Anastasios Potiris, Chrysi Christodoulaki, Athanasios Zachariou, Peter Drakakis, Konstantinos Zikopoulos, Ekaterini Domali, Athanasios Zikopoulos

**Affiliations:** 1 Laboratory of Medical Genetics, Faculty of Medicine, School of Health Sciences, University of Ioannina, 45110 Ioannina, Greece; 2 Third Department of Obstetrics and Gynecology, University General Hospital “ATTIKON”, Medical School, National and Kapodistrian University of Athens, 12462 Athens, Greece; sfstavrou@yahoo.com (S.S.); apotiris@gmail.com (A.P.); christodoulakichr@hotmail.com (C.C.); pdrakakis@med.uoa.gr (P.D.); thanzik92@gmail.com (A.Z.); 3 Department of Biological Applications & Technology, School of Health Sciences, University of Ioannina, 45110 Ioannina, Greece; tmichael@uoi.gr; 4 Department of Urology, School of Medicine, University of Ioannina, 45110 Ioannina, Greece; zahariou@otenet.gr; 5 Department of Obstetrics and Gynecology, Medical School, University of Ioannina, 45110 Ioannina, Greece; 6 First Department of Obstetrics and Gynecology, Alexandra Hospital, Medical School, National and Kapodistrian University of Athens, 11528 Athens, Greece; kdomali@yahoo.gr

**Keywords:** long COVID, reproductive health, autoimmune diseases, multiple sclerosis, menstrual irregularities, fertility, inflammation, immune dysregulation

## Abstract

Concern over COVID-19’s long-term influence on women’s reproductive health is growing, with emerging research suggesting potential links to ovarian dysfunction, menstrual irregularities, fertility challenges, and adverse pregnancy outcomes. Post-viral immune dysregulation is linked to both the development and exacerbation of autoimmune diseases, including multiple sclerosis (MS). Long COVID has been associated with immunological dysfunction, hormonal imbalances, and chronic inflammation, all of which may worsen autoimmune disorders and reproductive health issues. Long COVID is characterized by symptoms persisting for weeks or months beyond the acute infection phase. There are indications that prolonged COVID may contribute to autoimmune disease development through mechanisms such as immune hyperactivation, molecular mimicry, and dysregulated cytokine responses. Although this research field is still emerging, growing evidence suggests that SARS-CoV-2 infection may have lasting effects on women’s health, highlighting the need for further studies into its underlying mechanisms and long-term clinical outcomes. This review compiles recent findings on the long-term impact of COVID-19 on women’s reproductive health and its potential association with autoimmune disorders, particularly MS.

## 1. Introduction

The SARS-CoV-2 virus triggered the COVID-19 pandemic, impacting the entire world and infecting millions. While the acute phase has been well researched, the long-term effects, particularly on women’s reproductive health, are only now becoming fully apparent. There is increasing evidence that after COVID-19 infection, women, especially those of reproductive age, may experience challenges with pregnancy and fertility, irregular menstruation, and ovarian dysfunction. These persistent symptoms, referred to as protracted COVID, can significantly affect a woman’s overall quality of life and reproductive health [1].

In addition to influencing reproductive health, long-term COVID is linked to multiple autoimmune disorders, especially in women. Research indicates that immune system disruptions caused by SARS-CoV-2 infection may trigger or worsen autoimmune diseases. MS is a long-term autoimmune neurological disorder that mainly affects women [2,3]. These post-viral autoimmune responses may stem from immune system overactivation, molecular mimicry, and chronic inflammation, although the precise mechanisms are not yet fully understood. Further research is crucial due to the complexity of these conditions and their potential long-term impact on women’s health. To develop effective clinical care approaches, it is crucial to understand how these factors influence the progression of autoimmune diseases and reproductive health. This review aims to summarize the most recent findings on the long-term effects of COVID-19 on women’s reproductive health and its possible connection to autoimmune diseases, such as MS, to shed light on the underlying mechanisms and guide future treatment strategies [4].

## 2. Methodology

This review included peer-reviewed articles published in English from 2019 to 2024 that specifically addressed the relationship between SARS-CoV-2 infection and autoimmune diseases, with a particular focus on multiple sclerosis (MS). We included studies that investigated the pathophysiological mechanisms through which COVID-19 may contribute to the onset or exacerbation of autoimmune conditions, such as immune hyperactivation, molecular mimicry, and chronic inflammation. Eligible studies consisted of original research articles, case reports, systematic reviews, and meta-analyses that explored these mechanisms or clinical outcomes. Additionally, studies needed to provide data on the biological impact of SARS-CoV-2 on autoimmune disease development or worsening.

Studies were excluded if they did not involve human subjects, focused on non-autoimmune diseases, or lacked clear data on the mechanisms related to COVID-19 and autoimmune diseases. We also excluded non-peer-reviewed literature, such as preprints, abstracts, and editorials, as well as articles not providing insights into immune dysregulation or the clinical manifestations of COVID-19 in the context of autoimmune diseases.

As a narrative review, this article does not present a statistical synthesis of findings but rather summarizes current literature and identifies emerging trends. Therefore, no original data analysis or meta-analysis is included.

### Search Strategy

A comprehensive literature search was conducted in the following electronic databases: PubMed, Google Scholar, and Web of Science. The following keywords were used to retrieve relevant articles: “SARS-CoV-2”, “COVID-19”, “autoimmune diseases”, “multiple sclerosis”, “immune hyperactivation”, “molecular mimicry”, “chronic inflammation”, “cytokine storm”, and “COVID-19 and autoimmune diseases”. The search was limited to studies published between [start year] and [end year], and only English-language articles were included.

## 3. Overview of COVID-19 and Its Long-Term Effects

COVID-19, caused by the SARS-CoV-2 virus, was first identified in December 2019 in Wuhan, China, and rapidly spread across the globe, resulting in a pandemic that drastically altered public health and daily life. While respiratory symptoms such as fever, cough, and shortness of breath were most common during the acute phase of the illness, it has become increasingly clear that the virus can lead to long-term health consequences. These prolonged symptoms, commonly referred to as “long COVID”, affect a significant number of individuals who have recovered from the initial infection [5,6].

Long COVID refers to a range of symptoms that persist for weeks or even months after the acute phase of the illness has resolved. These symptoms can vary widely and include fatigue, brain fog, shortness of breath, muscle and joint pain, difficulty concentrating, headaches, and sleep disturbances. The full spectrum of long COVID symptoms is still being explored, but it is becoming evident that the effects of the virus extend far beyond the immediate infection, leading to chronic health issues. Long COVID can impact a variety of organ systems, including the immunological, neurological, cardiovascular, and reproductive systems, resulting in a complex and multifaceted illness [7].

Many women, particularly those in their reproductive years, report experiencing persistent symptoms that significantly affect their daily lives, suggesting that women may be disproportionately impacted by long COVID. A recent study suggests that women may be more likely than men to develop long-term symptoms after COVID-19, raising concerns about gender-specific impacts of the virus. It has been proposed that hormonal differences, immune system responses, and vulnerabilities in women’s reproductive systems could contribute to this disparity, though further investigation is required to confirm these hypotheses [8].

Although the exact mechanisms behind COVID-19’s long-term effects on reproductive health remain unclear, these effects are becoming more apparent. Many women who have recovered from the virus have reported menstrual cycle irregularities, including changes in flow, length, and intensity, as well as missed periods or delayed cycles. In some cases, women have experienced fertility issues, including difficulty conceiving or maintaining a pregnancy. A recent report has noted an increased occurrence of pregnancy complications, such as preterm birth and miscarriage, in women who have had COVID-19, contributing to concerns about the virus’s long-term impact on reproductive health [9].

Furthermore, it has been suggested that previous or concomitant infections with other viruses such as Zika virus may have similar or additive effects on maternal and fetal outcomes, especially given their known immunological and neurotropic impacts [10]. These findings support the need to investigate how viral co-infections may contribute to respiratory and reproductive complications, including asthma during pregnancy.

In addition to viral influences, emerging studies highlight the potential of nutritional interventions, such as betaine and myo-inositol supplementation, in supporting maternal health. These compounds have shown beneficial effects on lung maturation, breast tissue density, and possibly in preventing respiratory complications in both mothers and infants [11]. Though not yet widely adopted in clinical protocols, such interventions could offer supportive strategies in pregnancies complicated by long COVID or other stressors.

The potential long-lasting effects of COVID-19 on women’s reproductive health are particularly concerning, as they may affect a woman’s ability to conceive, carry a pregnancy to term, or maintain overall reproductive health. Even though research on this topic is still in its early stages, it is becoming increasingly clear that COVID-19 may have significant, long-term implications for fertility and pregnancy outcomes. Understanding the underlying mechanisms driving these effects is crucial to developing targeted care strategies and providing women with the appropriate support [9,12].

As long COVID continues to be studied, healthcare providers are focusing on offering comprehensive care that addresses the wide-ranging effects of the virus, including its impact on reproductive health. Women who experience chronic COVID symptoms related to reproduction may need specialized care to help manage their symptoms, improve their quality of life, and optimize their reproductive health. Ongoing research and patient-centered care are essential to better understanding and addressing these long-term health issues [13].

## 4. Impact of COVID-19 on Women’s Reproductive Health

The impact of COVID-19 on menstruation, fertility, and pregnancy has emerged as a significant area of concern. SARS-CoV-2 has been detected in reproductive tissues of both males and females, raising questions about potential effects on reproductive health. Some studies have detected viral RNA in the ovaries, uterus, and other reproductive tissues, but its direct impact on women’s fertility remains uncertain. Although post-infection menstrual irregularities have been reported, there is currently no strong evidence that the virus causes irreversible infertility [14].

Reported menstrual changes include alterations in cycle length, flow intensity, missed or delayed periods, and disrupted hormonal balance. These abnormalities are believed to be caused by either the virus itself or the body’s immune response, which may disrupt ovarian function or hormonal balance.

Complications during pregnancy have also been linked to COVID-19. Compared to women without the virus, pregnant women infected with it are more likely to experience miscarriage, premature birth, and issues with fetal development. These outcomes may be due to several factors [15,16,17]. Studies suggest that the virus may affect embryonic growth by inflaming the placenta and limiting blood flow. Furthermore, COVID-19 infection may elevate the risk of complications, including preeclampsia, a condition marked by high blood pressure during pregnancy.

Another concern is the possibility of vertical transmission, where the virus is passed from mother to fetus [18]. Although research indicates this is uncommon, it may still occur, especially in severe infections. This has prompted further inquiry into the long-term effects of prenatal COVID-19 exposure on neonatal and childhood health [19].

Emerging data also suggest that COVID-19 may have prolonged effects on fertility and pregnancy outcomes. Some women recovering from the infection report persistent symptoms such as menstrual irregularities and hormonal disturbances, which may continue beyond the acute phase [9,20].

Additionally, concerns have been raised about the impact of COVID-19 on assisted reproductive technologies (ART), such as in vitro fertilization (IVF), as the pandemic has led to treatment disruptions for some patients. Monitoring the impact of COVID-19 on women’s reproductive health is still crucial as our knowledge of the virus develops. Additional research is necessary to gain a complete understanding of the virus’s impact on fertility, pregnancy, and long-term reproductive health. This will be especially important for those who have contracted the virus, as well as for individuals planning a pregnancy or seeking reproductive treatments in the post-pandemic period [21].

## 5. Mechanisms Behind Post-Viral Autoimmune Responses

The long-term effects of COVID-19 raise significant concerns, particularly its potential to trigger or worsen autoimmune diseases. In individuals with a genetic predisposition, viral infections can cause the immune system to mistakenly attack the body’s tissues, resulting in autoimmune disorders. These post-viral autoimmune reactions may be triggered by various factors, although the exact mechanisms behind the development of autoimmune diseases following viral infections are still being studied. The over-activation of the immune system, known as immunological dysregulation, is one potential reason. In its attempt to fight the virus, the immune system may become hyperactive, causing it to attack both the infection and healthy tissues. This heightened immune response could contribute to the development of autoimmune diseases, especially in individuals with a genetic predisposition. For example, in conditions such as rheumatoid arthritis or lupus, the immune system may attack the joints or other tissues. Similarly, this overactivation could provoke a comparable immune response in COVID-19 patients [22].

Elevated pro-inflammatory cytokines, such as interleukin-6 (IL-6) and interferon-gamma (IFN-γ), during SARS-CoV-2 infection are known to exacerbate autoimmune processes. These cytokines can potentially trigger relapses in autoimmune diseases like MS and disrupt hormonal regulation, which may contribute to menstrual irregularities observed in women post-infection [23]. Additionally, immune hyperactivation and inflammation may play a role in pregnancy complications, including miscarriage, preterm birth, and preeclampsia, commonly reported in pregnant women with COVID-19. The underlying mechanisms such as placental inflammation and disrupted blood flow can exacerbate these complications. This connection highlights the need for a better understanding of how immune dysregulation during SARS-CoV-2 infection directly impacts clinical outcomes across various autoimmune and reproductive conditions [24].

Molecular mimicry is another possible mechanism, in which the immune system mistakenly recognizes the body’s tissues as foreign due to similarities between human and viral proteins. The proteins on the surface of the SARS-CoV-2 virus, which causes COVID-19, are structurally similar to those found in human proteins. As a result, the immune system may inadvertently attack cells that resemble the virus. This process has been linked to autoimmune diseases such as Guillain-Barré syndrome, where the immune system targets the peripheral nervous system [25].

Additionally, autoimmune diseases are greatly affected by chronic inflammation, both in terms of their onset and exacerbation. As part of the body’s immune response to infections like COVID-19, inflammatory cytokines are produced. In some individuals, these cytokines can trigger chronic inflammation even after the infection has resolved. This ongoing inflammation may worsen existing conditions or contribute to the development of autoimmune diseases [26]. Conditions like Hashimoto’s thyroiditis, an autoimmune disease that affects the thyroid gland, are associated with chronic inflammation after a viral infection.

Molecular mimicry, persistent inflammation, and immune system overactivation are the three primary causes of the rise in autoimmune disorders after COVID-19. Understanding these pathways is crucial for identifying individuals at greater risk of developing autoimmune issues after a viral infection and for developing effective treatment strategies to prevent or manage these conditions [27] (Table 1).

## 6. Autoimmune Diseases and Their Link to COVID-19 in Women

Autoimmune diseases exhibit a higher prevalence in women than in men. Among the most commonly observed conditions are lupus, MS, and rheumatoid arthritis. Although hormonal and genetic variables are thought to be responsible for this gender gap, the precise underlying causes are yet unknown. After infection, women may be more susceptible to developing new autoimmune disorders or experiencing flare-ups of pre-existing ones [37].

COVID-19 has been shown to both trigger and worsen autoimmune diseases. Research indicates that women who have recovered from COVID-19 are more likely to experience exacerbations of autoimmune conditions, such as thyroid disorders, lupus, and rheumatoid arthritis [38].

The development of new autoimmune diseases has also been associated with COVID-19, as some women without a prior history of autoimmune conditions have been diagnosed with rheumatoid arthritis or Hashimoto’s thyroiditis following infection. This implies that in genetically predisposed people, the virus may cause autoimmune reactions. The long-term immune system dysregulation brought on by COVID-19 probably contributes significantly, even if the exact pathways are still unknown [39].

## 7. MS and COVID-19: The Connection

MS is a chronic condition in which the immune system improperly targets nerve fibers’ protective coverings in the central nervous system. This damage leads to inflammation, disrupts nerve communication, and results in progressive neurological decline. Consequently, individuals with MS may experience symptoms like fatigue, cognitive difficulties, vision issues, and muscle weakness. MS predominantly affects women of reproductive age and is believed to result from a combination of genetic, environmental, and hormonal factors [40].

Concerns about a possible link between COVID-19 and MS persist, as viral infections have historically been linked to the onset and progression of the disease. The immunological response induced by SARS-CoV-2 may hasten the progression of MS, as some COVID-19 patients have experienced new relapses or worsened symptoms. Systemic inflammation caused by COVID-19 can worsen myelin damage in individuals with MS, leading to an increase in neurological disability [41]. Additionally, post-viral immune dysregulation has been linked to prolonged neurological symptoms, suggesting that COVID-19 may worsen MS-related issues. Along with worsening symptoms in those already diagnosed, research suggests that COVID-19 could trigger the onset of MS in previously healthy individuals. Case reports and observational studies have observed neurological symptoms similar to MS in people with no prior history of the disease following SARS-CoV-2 infection [7,42].

According to the hypothesis, COVID-19 may cause an excessive immune response in genetically susceptible individuals, resulting in inflammation and nerve tissue injury. Additionally, elevated levels of pro-inflammatory cytokines, such as interleukin-6 (IL-6), tumor necrosis factor-alpha (TNF-α), and interferon-gamma (IFN-γ), may contribute to the demyelination process. Another hypothesis proposes that molecular mimicry may be involved, with the immune system mistakenly attacking the nervous system due to structural similarities between SARS-CoV-2 viral antigens and myelin proteins [43].

Numerous studies have investigated the risk of MS onset or relapse following COVID-19 infection. Research has suggested that MS patients who contract COVID-19 may experience a worsening of pre-existing symptoms or the development of new neurological manifestations, although robust evidence linking COVID-19 directly to increased relapse rates is still limited [44]. There is growing evidence suggesting that severe COVID-19 infections may exacerbate neuroinflammation in MS patients, potentially influencing disease progression, but more studies are required to establish a clear link [45,46]. There are also growing concerns about SARS-CoV-2 acting as a trigger for autoimmune demyelination. Several case reports have documented previously healthy individuals developing MS-like symptoms after COVID-19, including optic neuritis and spinal cord lesions. Moreover, MS patients face an elevated risk of severe COVID-19 outcomes and possible disease progression, partly due to immune system compromise [47]. This is especially true for those receiving immunosuppressive therapies such as rituximab or ocrelizumab, which target and deplete B-cells. These treatments may impair viral clearance, prolong disease duration, and reduce vaccine efficacy. Consequently, special considerations for COVID-19 prevention and management are necessary for this population. Close monitoring is essential, as pre-existing comorbidities, respiratory muscle weakness, and reduced mobility further increase the risk of complications [48].

Managing both MS and COVID-19 presents several clinical challenges. Patients recovering from COVID-19 should receive neurological evaluations to monitor for new MRI-detected lesions, cognitive decline, and potential relapses. To balance the need for autoimmune control with the risk of COVID-19 complications, treatment approaches for disease-modifying therapies may need to be modified. Some studies suggest that anti-inflammatory and neuroprotective treatments, such as cytokine inhibitors, monoclonal antibodies, and corticosteroids, may help manage post-COVID immune hyperactivation in MS patients. To minimize their risk of infection, MS patients should follow their vaccination schedules and take preventive precautions [49].

Further longitudinal studies are needed to fully understand the long-term neurological effects of SARS-CoV-2 infection, as evidence of a complex connection between COVID-19 and MS continues to emerge. A proactive, interdisciplinary approach remains essential for managing both the short-term and long-term effects until more is known [50].

## 8. Clinical Observations and Case Studies

Clinical observations and case studies have shed light on COVID-19’s long-term effects on autoimmune illnesses and reproductive health in women. Researchers have documented an increase in incidences of infertility, menstrual abnormalities, and autoimmune disease aggravation among women who tested positive for SARS-CoV-2. COVID-19’s ongoing immunological dysregulation appears to have both direct and indirect consequences for these disorders, resulting in chronic symptoms and, in some cases, the emergence of new diseases [8].

Several case studies have documented significant menstrual alterations in women following COVID-19, including amenorrhea (absence of menstruation), heavy or irregular bleeding, and prolonged cycles. These abnormalities have been linked to inflammatory reactions, hormone imbalances, and even ovarian dysfunction caused by SARS-CoV-2 infection. COVID-19 has also been associated with fertility issues, particularly in women who have pre-existing reproductive problems such as polycystic ovary syndrome (PCOS) and endometriosis [15]. Studies of women undergoing fertility treatments indicate that post-COVID patients have reduced ovarian reserve markers, including lower anti-Müllerian hormone (AMH) levels, which are vital for reproductive potential. Pregnant women with COVID-19 faced elevated risks of miscarriage, preterm labor, and placental abnormalities [51]. A case-control study discovered that pregnant women who had persistent long-term COVID symptoms were more likely to develop preeclampsia and gestational diabetes, implying that COVID-19 may contribute to systemic inflammation and negatively impact maternal health outcomes [52].

Women with pre-existing autoimmune conditions, such as lupus, rheumatoid arthritis, and Hashimoto’s thyroiditis, have also reported more frequent and severe disease flare-ups post-COVID. The post-viral immune hyperactivation seen in long COVID appears to be a key factor in triggering or worsening these conditions [53]. Recent studies have highlighted the impact of COVID-19 on autoimmune diseases. For example, case reports and observational studies have shown that individuals with pre-existing autoimmune thyroid diseases, such as Hashimoto’s thyroiditis and Graves’ disease, may experience exacerbations following SARS-CoV-2 infection, presenting with increased fatigue, thyroid dysfunction, and autoimmune flares [54,55]. Furthermore, even mild COVID-19 infections in patients with systemic lupus erythematosus (SLE) have been linked to persistent symptoms, including joint inflammation and neurological manifestations, possibly due to sustained immune dysregulation [56].

Additionally, cases of new-onset autoimmune diseases such as type 1 diabetes, autoimmune arthritis, and SLE have been reported following COVID-19 infection, suggesting that the virus might act as a trigger for latent autoimmunity in genetically predisposed individuals [57,58]. Neurological complications resembling those seen in MS have also been described. Case reports have documented patients developing symptoms such as optic neuritis, limb weakness, and ataxia after recovering from COVID-19, raising concerns about COVID-19-induced neuroinflammation [59].

Moreover, observational studies indicate that individuals with pre-existing MS may experience symptom exacerbation or relapse after COVID-19, especially if the infection induces a prolonged inflammatory state [46]. A large UK cohort study involving 5875 MS patients reported a higher prevalence of new or worsened neurological symptoms following COVID-19 compared to uninfected MS patients [60]. More recently, evidence suggests that severe COVID-19 infections in MS patients are associated with increased neuroinflammation, accelerating disease progression [41] (Table 2).

These clinical findings underline the importance of ongoing monitoring and long-term follow-up for post-COVID patients, particularly women and those with pre-existing autoimmune disorders. The overlapping symptoms of long-term COVID and autoimmune illnesses provide considerable diagnostic problems, necessitating a multidisciplinary approach to ensure proper diagnosis and care. As research into post-COVID immunological responses progresses, more work is needed to discover the precise mechanisms causing these long-term consequences and develop tailored therapeutic techniques to lessen their impact [61].

## 9. Challenges in Diagnosing and Managing Long-Term Effects

Distinguishing and treating the long-term impact of COVID-19. Many symptoms of long-term COVID, such as chronic fatigue, brain fog, joint pain, and muscle weakness, are similar to those of autoimmune disorders, making it difficult for healthcare providers to determine whether a patient is experiencing a flare-up of an existing condition or new symptoms caused by post-viral syndrome [65]. Women with autoimmune conditions such as MS, lupus, or rheumatoid arthritis are especially prone to diagnostic uncertainty, as many of their symptoms overlap with those associated with long COVID. This resemblance can result in misdiagnosis or delays in effective treatment, compromising patient outcomes [2,66].

The diagnostic process is further complicated by the lack of specific biomarkers to differentiate long COVID from autoimmune conditions. Markers such as elevated inflammatory cytokines, autoantibodies, and immune dysregulation patterns are indicative of immune activation, yet their non-specificity means they are frequently observed in both long COVID and autoimmune diseases. Moreover, many individuals with COVID show no detectable abnormalities in routine blood tests, further complicating the differentiation between chronic post-viral symptoms and underlying autoimmune conditions. The unpredictable nature of chronic COVID, characterized by changes in symptom intensity and duration, makes diagnosis difficult, and it sometimes takes months of clinical observation before a conclusive distinction can be determined [67,68].

The management of long COVID is further hindered by the absence of standardized treatment protocols. In contrast to established autoimmune disorders, for which immunosuppressive agents, biologics, and disease-modifying therapies are available, long COVID currently lacks an approved cure or standardized treatment approach. Current therapeutic strategies rely on symptom-specific interventions, such as anti-inflammatory medications, corticosteroids, pain relievers, and lifestyle changes, but these treatments are primarily empirical rather than evidence-based. Some patients have claimed symptom relief with low-dose naltrexone (LDN), antihistamines, and anticoagulants, although these treatments are still experimental and not commonly used as routine care [69,70].

Additionally, nutritional interventions have gained attention for their potential supportive role. Betaine and inositol supplementation, for example, have demonstrated benefits in breast tissue modulation and fetal lung maturation, which may have implications for maternal respiratory health and neonatal outcomes—areas of concern in long COVID cases involving pregnancy or postpartum recovery. Although these supplements are not yet part of standard protocols, they offer a promising adjunctive strategy worth further investigation [71].

Another significant concern is the scarcity of specialist extended COVID clinics and interdisciplinary care teams. Long COVID’s involvement of multiple organ systems often compels patients to seek care from several specialists, including neurologists, rheumatologists, cardiologists, endocrinologists, and gynecologists, which may contribute to fragmented and uncoordinated management. Poor coordination among specialists, combined with the fragmentation of symptoms across medical domains, frequently causes significant delays in providing comprehensive care. This issue is especially concerning for women experiencing both reproductive health complications and autoimmune symptoms, particularly during pregnancy. Studies show that pregnant women with high-risk profiles may face additional barriers to accessing coordinated care, and concerns about long COVID or vaccination can further complicate clinical management [72,73].

Furthermore, the psychological impact of long-term COVID should not be disregarded. Many patients experience debilitating exhaustion, cognitive impairment, and chronic pain, which can lead to worry, sadness, and a lower quality of life. The lack of established therapeutic options, combined with an uncertain prognosis, contributes significantly to patient frustration and hopelessness. Many individuals struggle to receive a formal diagnosis from medical specialists, and their symptoms are frequently misdiagnosed as psychosomatic or stress-related disorders rather than a genuine post-viral disease. This has resulted in a growing advocacy movement for improved recognition, research funding, and specialized healthcare services for long-term COVID patients [74].

Progress depends on deeper investigation into the mechanisms of long COVID, especially its potential involvement in autoimmune activation. The identification of biomarkers capable of distinguishing long COVID from other chronic disorders will require large-scale studies, which are essential for the development of personalized treatment approaches. Furthermore, the development of standardized clinical standards for the detection and management of extended COVID is critical to ensuring patients receive timely and successful treatment. Increasing access to integrated care models, uniting various specialists within a single cohesive treatment framework, will be vital in managing the multifaceted challenges of chronic COVID and its relationship with autoimmune diseases. In the interim, managing long COVID will primarily focus on symptom relief, necessitating a patient-centered, multidisciplinary approach to optimize outcomes for those affected [75].

## 10. Future Research Directions

Further research is needed as our understanding of long-term COVID and its impact on women’s health continues to evolve. While significant work has been made in identifying COVID-19’s acute effects, the long-term impacts on immunological function, reproductive health, and disease progression remain poorly understood. As research into the virus’s broader effects progresses, it becomes evident that long-term COVID may affect women in distinct and profound ways, underscoring the need for specialized research [76].

Understanding the processes underlying post-viral autoimmune illnesses, specifically how COVID-19 causes or worsens these conditions in women, is an important focus of future research. To better understand the long-term effects of SARS-CoV-2, additional research is crucial to explore its role in immunological dysregulation, molecular mimicry, chronic inflammation, and hormonal disruptions. Evaluating the immune system’s long-term response to the virus allows researchers to differentiate post-COVID immune dysfunction from classic autoimmune conditions. Large-scale genetic and immunological studies are essential to understand whether certain individuals, especially women, are genetically predisposed to new autoimmune disorders or exacerbated disease activity following COVID. In addition, longitudinal research is needed to explore whether early interventions, including immunomodulatory therapies or anti-inflammatory treatments, can reduce autoimmune complications in long COVID patients [77].

Further research into COVID-19’s long-term effects on menstrual health, fertility, and pregnancy is also necessary. Although early data indicate that SARS-CoV-2 infection may cause menstrual cycle irregularities and ovarian dysfunction, large-scale cohort studies are essential to clarify whether these disruptions are temporary or suggest long-term reproductive health concerns. Research should assess hormonal fluctuations, ovarian reserve markers (e.g., AMH levels), and pregnancy outcomes in post-COVID patients to establish a clearer connection between the virus and reproductive health disorders. Furthermore, studies must explore whether COVID-19 infection during pregnancy has lasting consequences for maternal and fetal health, including potential epigenetic changes that could affect offspring’s health in the long term [78].

Gender-specific research is key to understanding how COVID-19 uniquely affects women and developing clinical care strategies that meet their specific needs. Unique immunological responses, hormonal fluctuations, and metabolic abnormalities in women may influence the severity and duration of long-term COVID symptoms. To promote equitable healthcare interventions, sex inequalities in illness presentation, treatment response, and recovery trajectories must be thoroughly examined. Further research is essential to understand how socioeconomic, environmental, and psychological factors affect women’s recovery from long COVID, particularly among vulnerable populations, such as pregnant women, those with autoimmune diseases, and those with limited healthcare access [79].

In addition to clinical research, public health interventions should be established to enhance awareness, diagnosis, and management of long-term COVID in women. More research should be conducted on the efficacy of preventative strategies, immunization outcomes, and prospective treatment interventions, particularly in reproductive health and autoimmune problems. Future study may inquire into the establishment of specialized long-term COVID clinics, where multidisciplinary teams can provide integrated and tailored therapy [79,80].

Addressing the long-term effects of COVID-19 on women’s health demands a comprehensive, collaborative research effort. Focusing on these key study areas allows the scientific and medical communities to create more effective, evidence-based treatments and improve patient outcomes. Ensuring that women receive the best possible care during and post-pandemic will require continued investment in research, regulatory changes, and healthcare innovations that address their unique needs [81].

## 11. Conclusions

**Figure 1:**
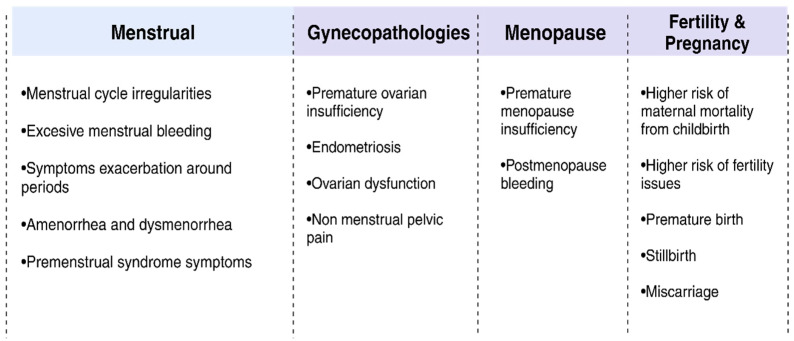
Summary of studies investigating the effects of COVID-19 on female reproductive health, organized into four primary domains: menstrual disturbances, gynecological pathologies, menopause-related symptoms, and fertility and pregnancy outcomes. This table summarizes the observed symptoms and complications reported within each category.

Conclusively, there is significant concern regarding the long-term effects of COVID-19 on women’s reproductive health (Figure 1), as well as its potential link to autoimmune diseases and MS. COVID-19 can have lasting impacts on women’s health, affecting everything from menstrual cycles and fertility to the onset or exacerbation of autoimmune disorders. To fully understand these implications, more research is needed. Women recovering from COVID-19 face distinct health challenges as our understanding of the virus’s long-term impacts continues to evolve, requiring specialized care. Improving the lives of those affected and mitigating these long-term effects will require advancements in healthcare practice and further research.

## Figures and Tables

**Table 1:** Proposed mechanisms by which SARS-CoV-2 infection may contribute to the development or worsening of autoimmune diseases, highlighting immune dysregulation, molecular mimicry, and chronic inflammation.

Mechanism	Description	Clinical Outcomes
Immune Hyperactivation	SARS-CoV-2 can cause immune system dysregulation, leading to chronic inflammation and potential autoimmune disease onset [21,28].	Increased disease flare-ups, progression of symptoms in MS and other autoimmune conditions.
Molecular mimicry	Viral proteins may resemble human proteins, triggering an immune response that mistakenly attacks the body’s own tissues [29,30].	New-onset or exacerbated autoimmune conditions, such as MS, due to immune system misfiring.
Chronic Inflammation	Persistent inflammation and cytokine dysregulation post-COVID-19 may contribute to autoimmune disease development or exacerbation [31,32].	Chronic inflammation can exacerbate MS symptoms and contribute to the development of other autoimmune disorders.
ACE2 Receptor-Mediated Entry	SARS-CoV-2 enters host cells via ACE2 receptors, which are found in multiple tissues, including the nervous system. This entry can lead to immune activation and autoimmunity, especially in individuals with genetic susceptibility [33,34].	Increased risk of neuroinflammation in MS and potentially other neurological autoimmune disorders.
Endothelial Dysfunction and Coagulopathy	COVID-19-induced damage to blood vessel linings (endothelial cells) and clot formation can worsen vascular health, leading to increased autoimmune flare-ups [35,36].	Exacerbation of symptoms in MS and other conditions involving vascular inflammation.

**Table 2:** Summary of key studies investigating the impact of COVID-19 on MS patients, including sample sizes, main findings regarding disease activity and relapses, and noted limitations of each study.

Study (Authors)	Sample Size	Key Findings	Limitations
Garjani et al. (2021) [60]	5875 participants from the UK MS Register; 657 reported suspected or confirmed COVID-19	COVID-19 was associated with a higher incidence of new MS symptoms Disease Modifying Therapies (DMTs) reduced the risk of new MS symptoms Ocrelizumab was the only DMT significantly linked to new MS symptoms during COVID-19	Self-reported data may introduce recall bias Some COVID-19 cases were based on symptoms rather than confirmed tests Findings may not be generalizable beyond the UK MS Register population
Babtain et al. (2022) [61]	301 patients 72% women; mean age 38 years Mean disease duration 10 years Median EDSS score 0.5	25% lower during the pandemic (26% vs. 51% pre-pandemic) Younger than 35, on disease-modify therapy (DMT), compliant with therapy 10% (30 patients) infected with COVID-19, all with mild symptoms, no hospitalizations COVID-19 impact → No association with clinical relapses or MRI changes Multivariate analysis → confirmed no effect of COVID-19 on disease activities	Retrospective design without a study protocol to control follow-up, potentially affecting the accuracy of disease activity estimates. Unequal follow-up periods before (3 years) and during pandemic (19 months) influencing disease activity Limited samples of 30 COVID-19 cases, which may not be enough to draw robust conclusions
Levitz et al. (2024) [62]	2253 cases 6441 controls; after matching 2161 cases and equal number of controls ∙	Cases (ARR = 0.10) had higher risk relapse rates than controls (ARR = 0.07) Cases had a significantly higher risk (HR = 1.54) compared to controls 24-week EDSS Progression → No association with COVID-19 infection (HR = 1.18) Subgroup (BRACE Therapy): COVID-19 infection increased the hazard of time to 1st relapse time to EDSS 3 (HR = 2.04)	Lack of PCR sequencing → no analysis of COVID-19 strain types Inconsistent vaccine data across sites prevented assessment of the vaccine’s relapse and disability Not accounting for key confounders like comorbidities, frailty, or body composition Short follow-up period of 1.7 years, limiting long-term conclusions Absence of data on COVID-19 severity and relapse duration
Prosperini et al. (2024) [63]	40 MS patients with confirmed COVID-19. Patients classified based on the severity of their COVID-19 course 19 mild 15 moderate 6 severe	47.5% of MS patients had mild COVID-19 courses, 37.5% moderate and 15% severe Older age and progressive MS phenotype were associated with severe courses Severe COVID-19 cases showed higher disability (60% moderate and 33.3% severe) DMT use did not differ across severity Patients with severe courses were more likely to be older, have a progressive MS phenotype, and exhibit significant disability	Small sample size, preventing proper risk-stratification and adjustment for confounders Significant discrepancy in sample size across disease severity groups, with only 6 patients in the severe group Potential for undetected differences in post hoc comparisons due to small sample sizes Larger studies are needed to better account for confounding variables and to validate findings
Salter et al. (2025) [64]	67.3%, 4787 participants completing COVID-19 questions	2106 participants reported confirmed COVID-19, with 796 having ≥3 surveys pre- and post-infection 1534 participants with no recent infections, 1336 had requisite surveys Minimal change in SMSS scores over time in both cohorts; no significant difference before or after COVID-19 infection Similar findings for disability outcomes cohorts between the COVID-19 and uninfected	Participants in the NARCOMS registry are volunteers and may not represent the general MS population While large, the sample size may still lack statistical power for meaningful differences The study used participant-reported scales, which may not detect changes as well as clinician assessments COVID-19 testing issues, inaccurate and undiagnosed infections may have occurred Uncontrolled COVID-19 severity

## Data Availability

No new data were created or analyzed in this study.
